# Preliminary assessment of anti-α-Gal IgG and IgM levels in patients with patent *Plasmodium vivax* infection

**DOI:** 10.1590/0074-02760190145

**Published:** 2019-07-04

**Authors:** Zélia Barbosa de Almeida Coelho, Luiza Carvalho Mourão, Beatriz Carolina Medeiros Rodrigues, Gustavo Pereira Cardoso-Oliveira, Robert Hincapie, Carlos Sanhueza-Chavez, MG Finn, Cor Jesus Fernandes Fontes, Alexandre Ferreira Marques, Érika Martins Braga

**Affiliations:** 1 Universidade Federal de Minas Gerais, Departamento de Parasitologia, Belo Horizonte, MG, Brasil; 2 Georgia Institute of Technology, School of Chemistry and Biochemistry, Atlanta, GA, USA; 3 Universidade Federal do Mato Grosso, Faculdade de Ciências Médicas, Cuiabá, MT, Brasil

**Keywords:** Plasmodium vivax, antibodies, α-Gal

## Abstract

Anti-α-Gal responses may exert a protective effect in falciparum malaria. However, the biological role of such antibodies is still unknown during *Plasmodium vivax* infections. We investigated IgG and IgM responses to α-Gal in individuals with vivax malaria. Anti-α-Gal IgG and IgM levels were higher in these patients than in controls, but no significant correlation was found between parasitaemia and anti-α-Gal response, nor between this response and ABO blood group status. This is the first study to investigate anti-α-Gal antibodies in *P. vivax*-infected patients; a larger survey is necessary to achieve a better understanding of host immune response during vivax malaria.

Anti-α-Gal antibodies are natural immunoglobulins present in high concentrations in human serum that recognise glycoconjugates (Gal α1,3Gal) on cell surface expressed on all mammalian cells, except old world monkeys, apes and humans. Since this glycan is not found in humans due to the inactivation of the enzyme α-1,3-galactosyltransferase (α-1,3GT),^1^ it is accepted that anti-α-Gal antibodies are generally produced in response to α-galactosyl epitopes expressed by bacteria of natural microbiotic fauna.^2^ ^,^ ^3^ Such antibodies may also be produced in response to infectious agents expressing α-Gal such as *Trypanosoma* spp and *Leishmania* spp.^4^ ^)^

Because anti-α-Gal antibodies have been implicated in the removal of senescent erythrocytes, as well as in different pathological phenomena including autoimmune and parasitic diseases,^3^ ^,^ ^5^ such immunoglobulins may be exploited for different beneficial clinical applications. Recently, Yilmaz et al.^6^ demonstrated that anti-α-Gal IgM antibodies are cytotoxic to *Plasmodium* sporozoites, inhibiting hepatocyte invasion. They also showed that immunisation against α-Gal confers protection against malaria in mice knockout for the gene α-1,3GT, suggesting that a similar approach could reduce malaria transmission in humans. Moreover a protective role for anti-α-Gal antibodies has been suggested by studies conducted with individuals from Mali^6^ and children from Senegal.^7^ In addition, elevated titres of anti-α-Gal antibodies have been detected in patients with acute *Plasmodium falciparum* infection.^8^ ^)^ Depending on the age of the child and the intensity of parasite exposure, the anti-αGal responses may vary. It has been demonstrated that anti-αGal IgM is protective, followed by IgG3 and IgG4 anti-αGal antibodies.^9^ ^)^ However, most of these studies have been conducted with patients with falciparum malaria; whether anti-α-gal antibodies confer protection to *Plasmodium vivax* infection remains unknown. Here, we assessed IgG and IgM antibody response to α-Gal in patients with patent *P. vivax* infection from Cuiabá, state of Mato Grosso, Brazil (n = 112) (Table), and as controls, malaria-naïve individuals who lived in a non-endemic area and who had never been exposed to malaria (Belo Horizonte, state of Minas Gerais, Brazil) (n = 20). This study was conducted according to the principles expressed in the Declaration of Helsinki and approved by the Ethics Committee of the National Information System on Research Ethics Involving Human Beings (Sisnep - CAAE01496013.8.0000.5149). All healthy donors and patients were anonymised, and they provided written informed consent for the collection of samples and subsequent analysis.

**TABLE:** Baseline characteristics of patients with acute *Plasmodium vivax* infection from the Brazilian Amazon (n = 112)

Parameter	Mean ± SD
Age (years)	37.7 ± 15.2
Number of malaria previous episodes	3.3 ± 4.4
Parasitaemia (parasites/µL)	5.531 ± 12..154
Haemoglobin (g/dL)	12.7 ± 2.8
Haematocrit (%)	38.4 ± 8.1
Platelets (cells/mm^3^)	124.353 ± 66.483
Leucocytes (cells/mm^3^)	5.471 ± 1.800

SD: standard deviation.

IgG and IgM anti-α-Gal were detected in plasma samples by enzyme-linked immunosorbent assay (ELISA) using 30 nm diameter bacteriophage Qβ virus like particles (Qβ-VLPs), displaying approximately 540 α-Gal molecules.^10^ Briefly, each well of a 96-well, flat-bottomed, polystyrene microplate (Corning Incorporation, Corning, NY, USA) was coated with 10 ng of Qβ-(αGal)_540_ particle in bicarbonate-carbonate buffer (pH 9.6; 0.1 M) and incubated overnight at 4ºC. Plates were blocked with 1% bovine serum albumin (BSA) in phosphate-buffered saline (PBS) pH 7.4 and kept at 37ºC for 1 hour; then, plasma samples diluted 1:100 in PBS/BSA were added and wells were incubated for 90 minutes at 37ºC. After three washes with PBS containing 0.05% (v/v) PBS Tween 20 (PBST), plates were incubated with biotinylated anti-human IgG or IgM, diluted, respectively, 1:4,000 and 1:5,000 in PBST for 30 minutes at 37ºC. Next, plates were rewashed and streptavidin-horseradish peroxidase (HRP) conjugate diluted 1:4,000 in PBST was added and maintained at 37ºC for 30 minutes. Binding was revealed using 0.5 mg/mL o-phenylenediamine dihydrochloride (OPD) substrate (Sigma-Aldrich, St Louis, MO, USA) in 0.05 M phosphate-citrate buffer, pH 5.0 and the reaction was stopped with 3 M H_2_SO_4_. Optical density (OD)_492_ nm was determined in a Spectra Max 250 microplate reader (Molecular Devices, Sunnyvale, CA, USA). Control measurements with underivatised Qβ particles showed no background binding by serum antibodies (data not shown).

*P. vivax* infection may increase anti-α-Gal IgG and IgM levels (Figure), data which are in accordance with previous findings for *P. falciparum*-infected patients.^8^ However, the magnitude of the anti-α-Gal responses were lower in patients with vivax malaria. Since anti-α-Gal antibodies are involved in different processes that contribute to allergies, such as tick-induced red meat allergy,^11^ ^,^ ^12^ autoimmune and “autoimmune-like” pathogeneses,^5^ ^,^ ^13^ ^)^ it is therefore possible that such antibodies may also play an important role in *P. vivax* infection. Although it has been demonstrated that IgG and IgM antibody responses to α-Gal vary according to age in *P. falciparum* infection,^9^ we found no significant age dependence in this study (Spearman’s correlation r = 0.2023, p = 0.0603 to IgG and r = -0.1339, p = 0.2163 to IgM). 

**Figure:**
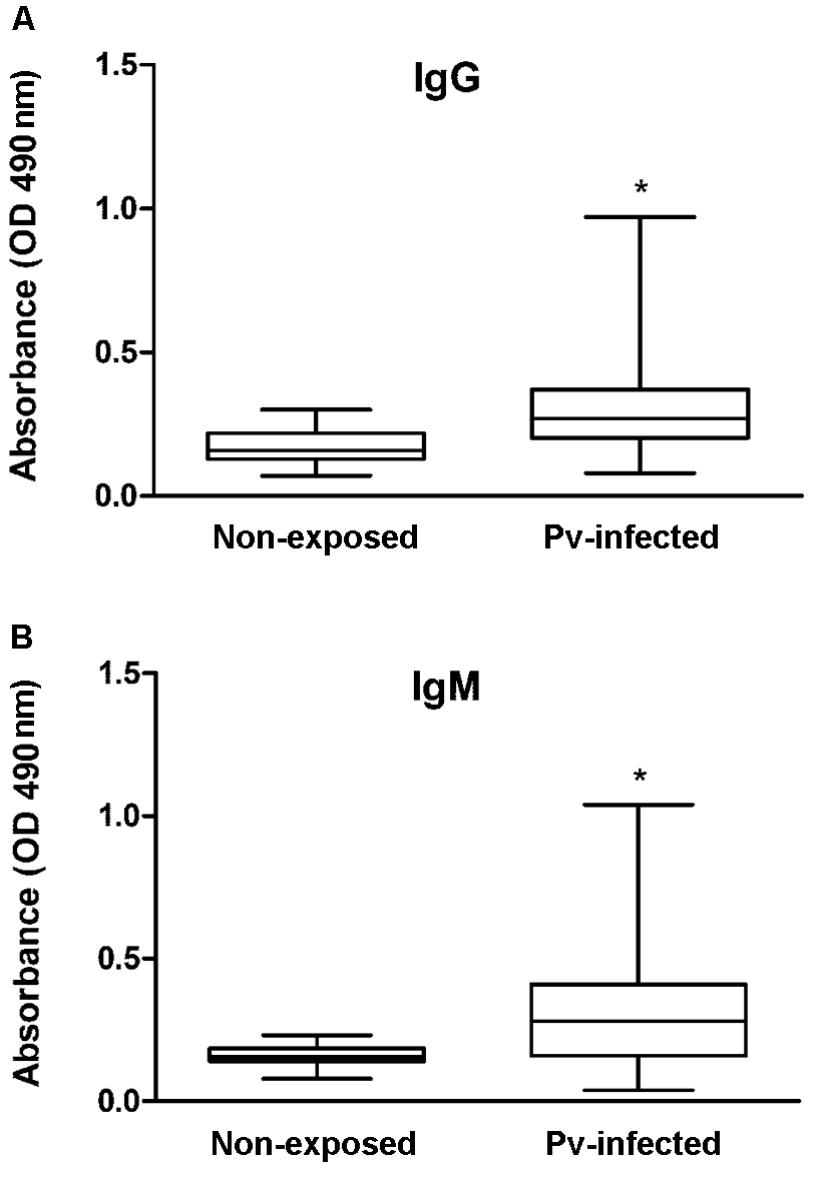
anti-α-Gal antibody responses in *Plasmodium vivax*-infected (Pv-infected) patients. Levels of IgG (A) and IgM (B) against α-Gal were evaluated in plasma from healthy individuals (n = 20) and patients with vivax malaria (n = 112) by enzyme linked immunosorbent assay (ELISA) and were expressed as values of optical density (OD). Results are shown as median values and interquartile ranges. Anti-α-Gal antibodies levels were compared between non-exposed and Pv-infected patients using Mann-Whitney *U* test and asterisks indicate statistically significant difference (p-value < 0.05).

We also evaluated the influence of parasitaemia, determined by examination of 200 fields at 1000x magnification under oil-immersion, on IgG and IgM responses to α-Gal. No significant correlation of parasitaemia with the levels of anti-α-Gal IgG or IgM was detected (Spearman’s correlation r = 0.2363, p = 0.0285 and r = 0.0291, p = 0.7899, respectively), but the scatter in the data is sufficiently large to suggest that a larger analysis is warranted. 

Taking into account the fact that the blood antigen B [Galα1-3(Fucα1,2)Gal] and the α-Gal glycan share a terminal Galα1-3 motif, and considering that individuals with blood type B have reduced antibody responses to α-Gal^14^ ^,^ ^15^ and are more susceptible to pathogens that express such antigen in their surface,^6^ ^,^ ^7^ ^)^ the effect of ABO blood type on immune responses to α-Gal in *P. vivax*-infected patients was also investigated. The ABO blood group was determined by reverse typing, testing each plasma (99 patients and 18 controls) (Supplementary Table) for the presence of anti-A and anti-B antibodies using known A and B erythrocytes (Revercel^®^, Fresenius Kabi, São Paulo, SP, Brazil). We did not found an association between anti-α-Gal IgG or IgM levels and blood type in subjects with patent *P. vivax* infection (Kruskal-Wallis followed by Dunn’s multiple comparison test p = 0.1740 and p = 0.2811, respectively).

Because IgG autoantibodies are involved in phagocytosis and complement-mediated cell lysis, establishing how well such immunoglobulins interact with the clearance system may provide valuable information to understand the host immune response during vivax malaria. To determine whether IgG anti-α-Gal was able to enhance innate clearance of non-infected erythrocytes from different blood groups, an assay was conducted as previously reported.^16^ Although we might expect B-type red blood cells to be less recognised by anti-α-Gal antibodies because of the molecular similarity of these two antigens, we observed no significant difference in erythrophagocytosis using erythrocytes from A, B or O blood groups. Such results suggest that opsonisation of non-infected erythrocytes by anti-α-Gal IgG is independent of ABO antigens, although it has already been demonstrated that differences in blood group antigen expression may affect susceptibility to other infections.^17^

Since early 1990s, glycoproteins and natural glycolipids have been shown to be important components of the adaptive immunological repertoire. Currently, there are no vaccines in use against more complex parasites such as *Plasmodium* spp, which leads to the necessity to identify antigens that are targets of naturally acquired antibodies against such important parasite. Further studies of anti-α-Gal response in different endemic populations are necessary to better elucidate the functional activity of anti-α-Gal antibodies in this context. 
