# Supplementary material for: Preliminary assessment of anti-α-Gal IgG and IgM levels in patients with patent Plasmodium vivax infection
Source: Mem Inst Oswaldo Cruz. 2019 Jul 4;114:e190145. doi: 10.1590/0074-02760190145 (PMC6611334; doi:10.1590/0074-02760190145)
Supplement: Supplementary file 1 [file 1678-8060-mioc-114-e190145-s.pdf]

TABLE
ABO blood type and anti-α-Gal antibody responses for each patient with patent *Plasmodium vivax* infection

Patient	Blood group	Anti-α-Gal IgG OD	Anti-α-Gal IgM OD
1	O	0.18	0.42
2	O	0.47	0.20
3	O	0.37	0.28
4	AB	0.24	0.30
5	B	0.22	0.19
6	O	0.36	0.29
7	A	0.48	0.57
8	A	0.48	0.37
9	O	0.38	0.25
10	B	0.25	0.36
11	B	0.22	0.20
12	O	0.20	0.32
13	B	0.23	0.18
14	AB	0.25	0.19
15	O	0.23	0.19
16	AB	0.31	0.20
17	O	0.21	0.21
18	B	0.22	0.23
19	O	0.38	0.23
20	O	0.20	0.20
21	A	0.24	0.23
22	O	0.24	0.33
23	O	0.13	0.22
24	O	0.10	0.23
25	A	0.31	0.28
26	A	0.23	0.16
27	A	0.26	0.23
28	O	0.22	0.29
29	AB	0.10	0.17
30	B	0.12	0.20
31	B	0.08	0.34
32	B	0.15	0.24
33	B	0.32	0.14
34	B	0.15	0.23
35	AB	0.23	0.22
36	B	0.45	0.51
37	O	0.29	0.27
38	O	0.34	0.34
39	B	0.43	0.58
40	O	0.32	0.33
41	B	0.25	0.39
42	O	0.63	0.39
43	B	0.28	0.54
44	A	0.35	0.30
45	O	0.24	0.38
46	B	0.20	0.41
47	B	0.22	0.49
48	B	0.28	0.35
49	O	0.27	0.34
50	O	0.19	0.48
51	A	0.43	0.38
52	O	0.22	0.50
53	O	0.16	0.57
54	O	0.19	0.41
55	A	0.18	0.41
56	B	0.25	0.23
57	O	0.13	0.49
58	O	0.20	0.37
59	O	0.30	0.34
60	B	0.34	0.28
61	B	0.22	0.39
62	B	0.23	0.37
63	B	0.35	0.25
64	AB	0.30	0.18
65	A	0.48	0.11
66	O	0.62	0.97
67	A	0.48	1.14
68	A	0.84	0.86
69	O	0.31	0.97
70	O	0.97	1.07
71	O	0.17	0.67
72	A	0.26	0.53
73	B	0.12	0.99
74	A	0.48	0.77
75	O	0.67	0.73
76	AB	0.34	0.44
77	O	0.41	0.79
78	B	0.62	0.65
79	O	0.52	0.79
80	A	0.21	0.28
81	O	0.29	0.36
82	O	0.32	0.53
83	A	0.22	0.47
84	B	0.20	0.41
85	B	0.52	0.32
86	A	0.27	0.46
87	AB	0.35	0.51
88	B	0.20	0.43
89	A	0.35	0.70
90	A	0.37	0.61
91	B	0.33	0.48
92	AB	0.17	0.23
93	A	0.17	0.54
94	B	0.15	0.32
95	A	0.18	0.48
96	O	0.29	0.53
97	O	0.42	0.48
98	B	0.43	0.39
99	B	0.30	0.26

Levels of IgG and IgM were detected by enzyme linked immunosorbent assay (ELISA) and expressed as values of optical density (OD).

CC BY
